# Non-Medical Risk Factors Influencing Health and Association with Suicidal Ideation or Attempt, U.S. Active Component, 2018–2022

**Published:** 2025-03-20

**Authors:** Evan Finlay, Saixia Ying, Sithembile L. Mabila, Shauna L. Stahlman

**Affiliations:** 1 Uniformed Services University of the Health Sciences, Bethesda, MD; 2 Epidemiology and Analysis Branch, Armed Forces Health Surveillance Division, Public Health Directorate, Defense Health Agency, Silver Spring, MD

## Abstract

This study reports the prevalence of non-medical risk factors, also known as social determinants of health, among active component service members and assesses the relationship between these factors and suicide ideation or attempts between 2018 and 2022. This analysis was performed to determine if there is opportunity to prevent suicide ideation or attempt among service members indicated for these non-medical risk factors. The findings reveal differences between demographic variables, emphasizing the disproportionate impacts of non-medical risk factors within the military population. For example, non-Hispanic Black service members had higher frequencies of diagnoses for all factors. After controlling for age, sex, service branch, race, and year of entry into military service, odds of suicidal ideation or attempt were elevated for service members with a recent diagnosis for factors related to abuse (odds ratio [OR] 13.7), family upbringing (OR 10.9), other psychosocial issues (OR 7.5), social environment (OR 7.4), lifestyle (OR 5.4), and life management (OR 5.3). This finding persisted even after excluding individuals with any prior mental health diagnosis. The results of this study suggest a need for a more comprehensive understanding of non-medical risk factors in shaping health outcomes and informing interventions to mitigate their effects.

**What are the new findings?:**

This study documents, for the first time, the frequency of diagnosis for non-medical risk factors influencing health among U.S. active component service members. An association is identified between non-medical risk factors and suicide ideation or attempt within 1 year following diagnosis of the risk factor.

**What is the impact on readiness and force health protection?:**

Suicide prevention is an aim of each military service. This study emphasizes the need for targeted interventions that address non-medical risk factors affecting health, to reduce mental health issues and suicide rates among service members. Improving access to resources and strengthening social support networks, to address issues related to abuse as well as economics, may enhance overall well-being and military readiness.

## 1 BACKGROUND

Non-medical risk factors that may influence health, also known as social determinants of health, pertain to the circumstances into which individuals are born, develop, reside, labor, and age, encompassing a broad spectrum of influences and systems that constitute daily living.^1^ In this article, the phrase “non-medical risk factors” is used instead of “social determinants of health,” as it is less fatalistic and more accurate in its description.

Non-medical factors including economic stability, education, neighborhood conditions, and access to health care play a significant role in shaping mental health and suicide outcomes in the U.S.^2^ Suicide remains a major public health crisis, with over 49,000 deaths, and 13.2 million individuals seriously considering suicide, in 2022, making it the ninth leading cause of death among people ages 10-64 years.^3^ Those facing financial hardship or housing instability are at an increased risk for mental illness and suicidal behaviors.^4,5^ According to one meta-analysis, the strongest risk factors for suicide attempts include childhood abuse and maltreatment, sexual assault, sexual minority status, and parental suicide mortality.^6^

While U.S. service members have benefits such as steady employment, housing allowance, and accessible health care, they are also affected by non-medical factors influencing health. Prior studies have indicated that factors such as familial problems can have independent associations with adverse outcomes such as suicide and medical evacuation from overseas deployments.^7,8^ The Army Study to Assess Risk and Resilience in Servicemembers (Army STARRS) found that childhood maltreatment and exposure to bullying was strongly associated with suicidal behaviors.^9,10^

Suicide is currently the leading cause of death for U.S. service members, and over 30,000 service members and veterans have died from suicide since September 11, 2001.^11^ Among active component service members (ACSMs) who attempted suicide in 2023, 33% had intimate relationship problems, 20% had workplace difficulties, and 9% experienced assault or harassment.^12^

While traditional risk factors such as combat exposure and deployment-related stressors have been studied extensively, there is a growing recognition that the broader context of non-medical risk factors plays a crucial role in shaping the mental health outcomes of military personnel.^13,14^ Understanding these non-medical risk factors is essential for identifying vulnerable subgroups within the active duty population and implementing interventions that address the broader contextual factors influencing suicide ideation and attempts. The objectives of this study were to 1) report the percentage of ACSMs diagnosed with non-medical factors influencing health in 2022 and 2) assess the relationship of non-medical factors influencing health with suicide ideation or attempt between 2018 and 2022.

## 2 METHODS

The surveillance case definition for non-medical factors influencing health were developed in 2023 through a Health Surveillance and Epidemiology Behavioral Health Working Group within Defense Health Agency Public Health.^15,16^ The International Classification of Diseases, 9th and 10th Revisions, Clinical Modification (ICD-9-CM and ICD-10-CM) code sets were based, in part, on code sets developed by the World Health Organization and the Centers for Disease Control and Prevention to monitor non-medical risk factors that may influence health, and were also based on a Veterans Administration (VA) study that looked at the effects of adverse social risk factors and their association with suicide risk and morbidity.^17^ The review group reviewed and modified the code sets to make them more relevant to service members and beneficiaries of the Military Health System and to behavioral health. ICD-9-CM codes were included because they were still being utilized in the Theater Medical Data Store (TMDS). Many of the factors are self-explanatory (e.g., alcohol and drug counseling, assault victim). It is worth mentioning, however, that the “life management” factor consisted of diagnoses like stress and “problems related to life management difficulty” and the “lifestyle” factor consisted of diagnoses such as inadequate sleep hygiene and “problems related to lifestyle.”

The data compiled for this study came from the Defense Medical Surveillance System (DMSS), a central repository of medical surveillance data for the U.S. Armed Forces. ACSMs diagnosed with non-medical factors influencing health were identified by having an inpatient, outpatient, or TMDS encounter with a qualifying ICD-9-CM or ICD-10-CM diagnosis in any diagnostic position.

For the first study objective, percentages were calculated as the number of ACSMs presenting with a non-medical factor influencing health in 2022 divided by the mid-year population size. Covariates included sex, age, race and ethnicity, service branch, rank, education, marital status, deployment history, and history of mental health diagnosis (ICD-9-CM: 290*-319*; ICD-10-CM: F*). A service member was counted only once for each factor. The total number and frequency of specific ICD-9-CM and ICD-10-CM diagnoses for non-medical factors influencing health in 2022 were also evaluated.

For the second study objective, a case-control study design was used to assess the relationship of past year diagnosis of non-medical factors influencing health with suicide ideation or attempt. Suicide ideation and attempt were combined into a single variable because, although these conditions can have different risk factors, many risk factors are also shared, and it is possible to attempt suicide without reporting prior ideation.^18^ Incident (i.e., first-ever diagnosis since joining military service) cases of suicidal ideation or attempt were identified by an inpatient, outpatient, or TMDS encounter between 2018 and 2022 with a qualifying diagnosis (ICD-9-CM: V62.84, E958.9; ICD-10-CM: R45.851, T14.91*) in any diagnostic position. Each incident case was matched to up to 3 controls on year of birth, sex, race, service branch, and year of entry into military service. Controls were required to be in service at the time of the case diagnosis and to have no qualifying suicidal ideation or attempt diagnoses on or prior to December 31, 2022. In a secondary analysis, the case-control study was repeated on a population of ACSMs who had no history of mental health diagnoses on or prior to December 31, 2022. Conditional logistic regression was used to calculate odds ratios and 95% confidence intervals. All analyses were performed using SAS® Enterprise Guide® software (version 8.3, SAS Inst Inc, Cary, NC).

## 3 RESULTS

**Period prevalence for non-medical risk factors**

In 2022 there were 634,233 diagnoses of non-medical risk factors among 161,668 ACSMs (data not shown). The percentage and number of service members diagnosed for each factor are shown in **Table 1**. A service member could have multiple diagnoses for each factor, and the most common diagnoses for each factor are shown in **Table 2**. The most commonly diagnosed factor was Family and Upbringing (179,370 diagnoses among 49,381 individuals). The most common diagnoses within the Family and Upbringing factor were ‘Problems in relationship with spouse or partner’ (55% of total diagnoses), ‘Disappearance and death of family member’ (15%), and ‘Problems related to primary support group’ (9%) (**Table 2**). Employment was the second-most commonly diagnosed factor (98,159 diagnoses among 36,803 individuals), and Other Psychosocial was the third-most commonly diagnosed (95,474 diagnoses among 34,920 individuals). The Physical Environment factor only included 4 diagnoses of Z586 “Inadequate drinking-water supply” among 3 individuals, so it was excluded from further analysis.

After inspection of the data, it appeared that health care providers were diagnosing the Perpetrator of Violence factor in the medical encounters for both victims and perpetrators. Of the 196 diagnoses in 2022 for ICD-10-CM code Y0701 ‘Husband, perpetrator of maltreatment and neglect’, 141 (72%) were listed in male encounters and 55 (28%) were listed in female encounters. Among female encounters, 34 (62%) also had an injury diagnosis (ICD-10-CM diagnosis beginning with ‘S’ or ‘T’), suggesting these were victims of violence. Among the male encounters, almost none had an injury diagnosis, and 63 (45%) had a counseling diagnosis beginning with Z71, suggesting these were perpetrators of violence.

Non-Hispanic Black service members were more frequently diagnosed for all non-medical risk factors compared to other racial and ethnic groups, with the exception of the Education and Literacy factor, which was similar for all groups (**Table 1**). Similarly, ACSMs with a prior diagnosis of depression, anxiety, and post-traumatic stress disorder (PTSD) had a higher prevalence of non-medical risk factor diagnosis. Women, enlisted members, and those with less education also had higher percentages of diagnoses with many non-medical risk factors as compared to men, officers, and those with higher education levels. Married service members had lower prevalence of some factors (e.g., employment, alcohol and drug counseling, and lifestyle) compared to single, never-married members. The percentage diagnosed with Family and Upbringing and Life Management factors increased with increasing age. In contrast, those younger than age 20 years had the highest prevalence of Employment factor diagnosis. The Army had the highest prevalence of non-medical risk factor diagnosis for all factors except Life Management (which was highest among Air Force members) and Alcohol and Drug Counseling (highest among Navy members).

**Suicide ideation or attempt and non-medical risk factors**

There were 85,962 cases and 242,763 matched controls identified to assess the relationship between suicide ideation or attempt and non-medical risk factors diagnosed in the preceding year (data not shown). Of the identified cases, 95% were diagnosed with suicide ideation and 5% were diagnosed with suicide attempt. A total of 42,672 (49.6%) cases had a diagnosis for any non-medical risk factor within a year preceding incident diagnosis, compared to 18,921 (7.8%) controls. For cases, the average (mean) number of days between non-medical factor diagnosis and incident suicide ideation or attempt was 66 days, and the median was 203 days.

After controlling for year of birth, sex, race, branch of military service, and year of entry into service, there was a statistically significant positive association between past year diagnosis of all non-medical risk factors and diagnosis of suicide ideation or attempt (**Table 3**). Odds of suicidal ideation or attempt were highest for Housing and Economics (odds ratio [OR] 27.3), followed by Physical, Sexual and Psychological Abuse (OR 13.7), Employment (OR 12.9), and Family and Upbringing (OR 10.9) factors.

A secondary analysis calculated the odds of suicidal ideation or attempt among service members without prior mental health diagnoses, using the same matching factors as the primary analysis. After exclusions, 3,204 cases were matched to 9,239 controls. Among service members with no prior mental health diagnoses, there was a statistically significant positive association between past year diagnosis of Employment (OR 56.0), Social Environment (OR 35.9), Life Management (OR 16.6), Family Upbringing (OR 15.5), Other Psychosocial (OR 11.5), Physical, Sexual and Psychological Abuse (OR 8.3), and Lifestyle (OR 4.17), factor with diagnosis of suicide ideation or attempt (**Table 4**).

A sensitivity analysis was conducted to determine whether adjustment for deployment history or marital status would change the odds ratio estimates in both the primary and secondary logistic regression analyses. No significant deviations from the original odds ratio estimates were observed, suggesting that deployment history and marital status were not significant confounders in these associations.

## 4 DISCUSSION

The study documents, for the first time, the frequency of diagnosis for non-medical risk factors influencing health among ACSMs. Notably, non-Hispanic Black individuals consistently exhibited the highest percentages in all risk categories, highlighting the disproportionate burden they face. This finding is consistent with other research within the U.S. population showing that the non-Hispanic Black population has less economic security and more problems associated with family and upbringing.^19,20^

Despite facing greater social adversity, non-Hispanic Black service members exhibit lower rates of suicidal ideation and attempts.^21^ Future research should explore the protective factors, such as cultural or social resilience, that may contribute to this trend. Additionally, studies should assess whether non-medical risk factor codes accurately capture suicide risk across different racial groups. Addressing these gaps could enhance suicide prevention efforts and improve risk assessment strategies.

Age proved interesting, as those trends were not consistent for all factors. Categories such as Family and Upbringing and Life Management demonstrated a percentage increase with increase in age, while non-medical factors affecting Employment and Lifestyle saw inverse effects with increasing age. Similar findings are demonstrated within the U.S. population for employment factors, as those who are older tend to find more fulfillment and less likelihood of feeling overwhelmed compared to their younger counterparts.^22^ The vast majority of Life Management diagnoses (89%) in this study were for ‘Stress, not elsewhere classified’, the opposite of what is observed in the U.S. population, where stress levels typically decrease as individuals age.^23^ This could be due to unique military experiences such as deployments, change in duty station, or combat exposures. Additional military-specific research should, however, investigate these age-related trends.

This analysis further revealed that individuals with diagnoses of certain factors were at heightened risk for suicide ideation or attempt both before and after excluding those with prior histories of mental health diagnoses. This finding is consistent with findings from broader U.S. population studies and studies conducted in veterans,^17,24,25^ suggesting that certain non-medical factors, including elements of family background, upbringing, prior trauma, and adverse life experiences, are critical considerations, as they may contribute to suicidal behaviors long before individuals enlist in the military. This finding also presents an opportunity for possible interventions, particularly for service members presenting with these factors but not already engaged in mental health treatment.

Limitations to this study include variability in coding practices among providers or coders. The use of ICD-9-CM and ICD-10-CM diagnoses to identify both factors and suicidal ideation and attempt outcomes likely led to the under-capture of both exposures and outcomes. It is also possible that those with a factor diagnosis may be followed more closely by their providers for suicidal ideation or attempt, which could contribute towards the associations observed in this study. Additional analyses such as by using self-reported mental health and suicidal ideation from Periodic Health Assessments could help to confirm the associations reported in this study.

Future studies could also consider investigating additional comorbidities associated with these factors, such as obesity and stroke,^26,27^ as well as the compound effects of multiple non-medical risk factors. Service members should be encouraged to report any non-medical factors influencing health so interventions can be targeted, and so that more complete data exist on the magnitude of issues. Understanding the effects of non-medical risk factors on medical conditions, like suicide ideation or attempt, can hopefully mitigate their effects and decrease their prevalence within the Military Health System.

## Figures and Tables

**Table 1:** Active Component U.S. Service Members Diagnosed with Non-Medical Factors Influencing Health, 2022

	Education and Literacy		Employment		Housing and Economic		Social Environment		Family and Upbringing		Other Psychosocial		Sexual Behavior Counseling		Alcohol and Drug Counseling		Lifestyle		Life Management		Physical, Sexual and Psychological Abuse Victim		Assault Victim		Perpetrator of Violence	
	No.	%	No.	%	No.	%	No.	%	No.	%	No.	%	No.	%	No.	%	No.	%	No.	%	No.	%	No.	%	No.	%
Total	236	0.02	36,803	2.84	1,141	0.09	19,255	1.48	49,381	3.81	34,920	2.69	785	0.06	4,185	0.32	22,696	1.75	31,453	2.42	20,151	1.55	2,329	0.18	251	0.02
Sex																										
Male	164	0.02	27,299	2.55	893	0.08	14,012	1.31	35,545	3.32	26,147	2.44	572	0.05	3,575	0.33	18,347	1.71	22,164	2.07	10,830	1.01	1,776	0.17	102	0.01
Female	72	0.03	9,504	4.19	248	0.11	5,243	2.31	13,836	6.10	8,773	3.87	213	0.09	610	0.27	4,349	1.92	9,289	4.10	9,321	4.11	553	0.24	149	0.07
Age, y																										
<20	42	0.05	3,131	3.78	59	0.07	1,776	2.14	1,331	1.61	1,600	1.93	29	0.03	257	0.31	1,061	1.28	1,262	1.52	1,311	1.58	209	0.25	20	0.02
20-24	82	0.02	13,376	3.22	476	0.11	6,527	1.57	14,286	3.44	12,947	3.12	317	0.08	1,722	0.41	9,269	2.23	9,161	2.21	8,332	2.01	1,216	0.29	107	0.03
25-29	56	0.02	8,788	2.90	299	0.10	4,137	1.37	12,311	4.07	8,772	2.90	239	0.08	1,083	0.36	5,664	1.87	7,559	2.50	4,894	1.62	510	0.17	59	0.02
30-34	31	0.01	5,061	2.41	175	0.08	2,321	1.10	8,502	4.04	5,029	2.39	126	0.06	515	0.24	2,949	1.40	4,837	2.30	2,584	1.23	207	0.10	31	0.01
35-39	11	0.01	3,608	2.26	69	0.04	1,915	1.20	7,117	4.46	3,720	2.33	45	0.03	377	0.24	2,110	1.32	4,434	2.78	1,808	1.13	114	0.07	24	0.02
40-44	8	0.01	1,881	2.32	32	0.04	1,559	1.92	3,876	4.77	1,948	2.40	24	0.03	176	0.22	1,091	1.34	2,649	3.26	850	1.05	50	0.06	7	0.01
45+	6	0.01	958	2.09	31	0.07	1,020	2.22	1,958	4.27	904	1.97	5	0.01	55	0.12	552	1.20	1,551	3.38	372	0.81	23	0.05	3	0.01
Race and ethnicity																										
White, non-Hispanic	114	0.02	16,883	2.43	472	0.07	9,381	1.35	22,643	3.26	15,622	2.25	278	0.04	1,990	0.29	9,846	1.42	14,806	2.13	9,127	1.31	1,043	0.15	93	0.01
Black, non-Hispanic	39	0.02	9,295	4.49	336	0.16	4,390	2.12	12,490	6.03	9,286	4.48	253	0.12	959	0.46	5,859	2.83	6,978	3.37	5,163	2.49	539	0.26	76	0.04
Hispanic	56	0.02	6,669	2.79	211	0.09	3,513	1.47	9,178	3.84	6,635	2.78	160	0.07	805	0.34	4,633	1.94	5,819	2.44	3,996	1.67	512	0.21	60	0.03
Other	22	0.02	3,521	2.55	111	0.08	1,704	1.24	4,469	3.24	3,058	2.22	77	0.06	362	0.26	2,101	1.52	3,353	2.43	1,654	1.20	210	0.15	19	0.01
Unknown	5	0.03	435	2.32	11	0.06	267	1.43	601	3.21	319	1.70	17	0.09	69	0.37	257	1.37	497	2.65	211	1.13	25	0.13	3	0.02
Service																										
Army	118	0.03	21,055	4.57	747	0.16	11,095	2.41	29,835	6.47	24,563	5.33	201	0.04	725	0.16	10,937	2.37	13,036	2.83	11,240	2.44	1,053	0.23	130	0.03
Navy	49	0.01	7,169	2.11	192	0.06	2,669	0.79	7,023	2.07	3,582	1.05	270	0.08	1,935	0.57	3,602	1.06	6,318	1.86	2,776	0.82	507	0.15	56	0.02
Air Force	46	0.01	5,593	1.74	134	0.04	4,221	1.31	9,932	3.08	5,176	1.61	288	0.09	1,315	0.41	5,029	1.56	9,700	3.01	4,930	1.53	316	0.10	56	0.02
Marine Corps	23	0.01	2,986	1.71	68	0.04	1,270	0.73	2,591	1.48	1,599	0.92	26	0.01	210	0.12	3,128	1.79	2,399	1.37	1,205	0.69	453	0.26	9	0.01
Rank/grade																										
Junior enlisted (E1-E4)	158	0.03	20,358	3.78	733	0.14	9,908	1.84	20,188	3.75	18,646	3.46	377	0.07	2,503	0.46	12,129	2.25	12,888	2.39	11,389	2.12	1,623	0.30	159	0.03
Senior enlisted (E5-E9)	46	0.01	12,741	2.43	353	0.07	7,241	1.38	23,298	4.45	13,200	2.52	293	0.06	1,417	0.27	8,387	1.60	14,188	2.71	7,331	1.40	579	0.11	79	0.02
Junior officer (O1-O3)	22	0.02	2,214	1.68	29	0.02	970	0.73	2,631	1.99	1,642	1.24	96	0.07	161	0.12	1,335	1.01	2,173	1.65	794	0.60	94	0.07	9	0.01
Senior officer (O4-O10)	9	0.01	1,076	1.28	15	0.02	845	1.01	2,330	2.78	920	1.10	17	0.02	80	0.10	610	0.73	1,813	2.16	428	0.51	20	0.02	4	0.00
Warrant officer (W*)	1	0.01	414	2.15	11	0.06	291	1.51	934	4.84	512	2.66	2	0.01	24	0.12	235	1.22	391	2.03	209	1.08	13	0.07		
Education level																										
High school or less	165	0.02	25,803	3.17	894	0.11	12,861	1.58	31,289	3.84	24,638	3.03	501	0.06	3,369	0.41	16,185	1.99	18,979	2.33	14,753	1.81	1,903	0.23	190	0.02
Some college	25	0.02	4,372	2.88	121	0.08	2,519	1.66	7,780	5.13	4,534	2.99	97	0.06	337	0.22	2,584	1.70	4,768	3.14	2,546	1.68	197	0.13	35	0.02
Bachelor's or advanced degree	40	0.01	6,264	2.07	115	0.04	3,676	1.21	9,734	3.21	5,431	1.79	157	0.05	417	0.14	3,635	1.20	7,247	2.39	2,702	0.89	205	0.07	25	0.01
Unknown	6	0.02	364	1.27	11	0.04	199	0.70	578	2.02	317	1.11	30	0.11	62	0.22	292	1.02	459	1.61	150	0.53	24	0.08	1	0.00
Marital status																										
Single, never married	151	0.03	17,969	3.11	480	0.08	8,774	1.52	12,081	2.09	14,676	2.54	521	0.09	2,365	0.41	12,252	2.12	11,563	2.00	8,184	1.42	1,395	0.24	102	0.02
Married	78	0.01	16,709	2.56	588	0.09	9,231	1.41	33,844	5.18	17,767	2.72	206	0.03	1,581	0.24	8,817	1.35	17,605	2.69	10,298	1.58	817	0.13	133	0.02
Other	7	0.01	2,125	3.19	73	0.11	1,250	1.88	3,456	5.19	2,477	3.72	58	0.09	239	0.36	1,627	2.44	2,285	3.43	1,669	2.50	117	0.18	16	0.02
Deployment history																										
Ever deployed	31	0.01	7,632	1.91	183	0.05	4,979	1.25	14,372	3.60	7,696	1.93	182	0.05	1,119	0.28	5,294	1.33	10,369	2.60	4,364	1.09	326	0.08	39	0.01
Never deployed	205	0.02	29,171	3.25	958	0.11	14,276	1.59	35,009	3.90	27,224	3.03	603	0.07	3,066	0.34	17,402	1.94	21,084	2.35	15,787	1.76	2,003	0.22	212	0.02
Any prior mental health diagnosis																										
Yes	125	0.03	24,085	4.98	841	0.17	12,162	2.52	35,445	7.34	23,318	4.83	357	0.07	2,759	0.57	12,742	2.64	19,901	4.12	14,885	3.08	1,091	0.23	154	0.03
No	111	0.01	12,718	1.56	300	0.04	7,093	0.87	13,936	1.71	11,602	1.42	428	0.05	1,426	0.18	9,954	1.22	11,552	1.42	5,266	0.65	1,238	0.15	97	0.01
Prior depressive disorder diagnosis																										
Yes	54	0.04	9,118	7.30	384	0.31	4,369	3.50	13,839	11.08	8,144	6.52	112	0.09	977	0.78	4,130	3.31	6,201	4.97	6,746	5.40	352	0.28	73	0.06
No	182	0.02	27,685	2.36	757	0.06	14,886	1.27	35,542	3.03	26,776	2.28	673	0.06	3,208	0.27	18,566	1.58	25,252	2.15	13,405	1.14	1,977	0.17	178	0.02
Prior anxiety disorder diagnosis																										
Yes	56	0.04	9,539	6.03	366	0.23	4,907	3.10	14,898	9.42	8,812	5.57	130	0.08	925	0.59	4,546	2.88	8,035	5.08	6,715	4.25	381	0.24	64	0.04
No	180	0.02	27,264	2.39	775	0.07	14,348	1.26	34,483	3.03	26,108	2.29	655	0.06	3,260	0.29	18,150	1.59	23,418	2.06	13,436	1.18	1,948	0.17	187	0.02
Prior PTSD diagnosis																										
Yes	14	0.03	2,880	5.97	125	0.26	1,494	3.09	5,289	10.95	2,846	5.89	43	0.09	325	0.67	1,452	3.01	2,310	4.78	3,271	6.78	171	0.35	32	0.07
No	222	0.02	33,923	2.72	1,016	0.08	17,761	1.42	44,092	3.53	32,074	2.57	742	0.06	3,860	0.31	21,244	1.70	29,143	2.33	16,880	1.35	2,158	0.17	219	0.02

Abbreviations: y, years; PTSD, post-traumatic stress disorder.

**Table 2:** Three Most Common Diagnoses for Each Non-Medical Factor, Total Diagnoses, Active Component U.S. Service Members, 2022^a^

ICD Code	ICD Code Description	No.	%
Education and literacy (n=438)			
Z559	Problems related to education and literacy, unspecified	267	61.0
Z558	Other problems related to education and literacy	101	23.1
Z552	Failed school examinations	53	12.1
Employment (n=98,159)			
Z5689	Other problems related to employment	48,765	49.7
Z569	Unspecified problems related to employment	20,669	21.1
Z566	Other physical and mental strain related to work	18,300	18.6
Housing, economic (n=2,348)			
Z5989	Other problems related to housing and economic circumstances	877	37.4
Z599	Problem related to housing and economic circumstances, unspecified	837	35.6
Z5900	Homelessness, unspecified	169	7.2
Social environment (n=45,191)			
Z600	Problems of adjustment to life cycle transitions	36,120	79.9
Z609	Problem related to social environment, unspecified	3,112	6.9
Z603	Acculturation difficulty	2,394	5.3
Family and upbringing (n=179,370)			
Z630	Problems in relationship with spouse or partner	99,122	55.3
Z634	Disappearance and death of family member	26,288	14.7
Z638	Other specified problems related to primary support group	15,547	8.7
Other psychosocial (n=95,474)			
Z658	Other problems related to psychosocial circumstances	58,221	61.0
Z659	Problem related to unspecified psychosocial circumstances	17,326	18.1
Z653	Problems related to other legal circumstances	13,322	14.0
Sexual behavior counseling (n=1,235)			
Z701	Counseling related to patient's sexual behavior and orientation	1,053	85.3
Z703	Counseling related to combined concerns regarding sexual attitude, behavior and orientation	105	8.5
Z700	Counseling related to sexual attitude	70	5.7
Alcohol, drug counseling (n=13,552)			
Z7141	Alcohol abuse counseling and surveillance of alcoholic	12,449	91.9
Z7151	Drug abuse counseling and surveillance of drug abuser	889	6.6
Z714	Alcohol abuse counseling and surveillance	110	0.8
Lifestyle (n=41,119)			
Z72821	Inadequate sleep hygiene	13,091	31.8
Z7289	Other problems related to lifestyle	11,908	29.0
Z7251	High risk heterosexual behavior	5,432	13.2
Life management (n=65,954)			
Z733	Stress, not elsewhere classified	59,059	89.5
Z7389	Other problems related to life management difficulty	3,947	6.0
Z739	Problem related to life management difficulty, unspecified	845	1.3
Victim of physical, sexual, psychological abuse (n=87,094)			
Z62810	Personal history of physical and sexual abuse in childhood	18,578	21.3
Z91410	Personal history of adult physical and sexual abuse	14,176	16.3
Z62811	Personal history of psychological abuse in childhood	7,394	8.5
Victim of assault (n=3,785)			
Y040XXA	Assault by unarmed brawl or fight, initial encounter	801	21.2
Y09	Assault by unspecified means	652	17.2
Y042XXA	Assault by strike against or bumped into by another person, initial encounter	588	15.5
Perpetrator of violence (n=510)			
Y0701	Husband, perpetrator of maltreatment and neglect	196	38.4
Y079	Unspecified perpetrator of maltreatment and neglect	130	25.5
Y0759	Other non-family member, perpetrator of maltreat and neglect	61	12.0

Abbreviations: ICD, International Classification of Diseases; n, number.

^a^Physical Environment factor only included 4 diagnoses of Z586 “Inadequate drinking-water supply” among 3 individuals, so it was excluded from further analysis.

**Table 3:** Adjusted Odds of Incident Suicidal Ideation or Attempt, Active Component U.S. Service Members, 2018–2022

	Cases	Controls				
	No.	%	No.	%	OR	95% LL	95% UL	*p*-value
Education and literacy^a^								
Yes	108	0.1	47	0.0	6.4	4.5	9.0	<.0001
No	85,854	99.9	242,716	100.0	Reference	–	–	
Employment^a^								
Yes	15,678	18.2	4,302	1.8	12.9	12.4	13.4	<.0001
No	70,284	81.8	238,461	98.2	Reference	–	–	
Housing, economic^a^								
Yes	1,123	1.3	122	0.1	27.3	22.5	33.1	<.0001
No	84,839	98.7	242,641	99.9	Reference	–	–	
Social environment^a^								
Yes	6,334	7.4	2,608	1.1	7.4	7.1	7.8	<.0001
No	79,628	92.6	240,155	98.9	Reference	–	–	
Family upbringing^a^								
Yes	16,708	19.4	5,931	2.4	10.9	10.5	11.2	<.0001
No	69,254	80.6	236,832	97.6	Reference	–	–	
Other psychosocial^a^								
Yes	9,623	11.2	4,392	1.8	7.5	7.2	7.8	<.0001
No	76,339	88.8	238,371	98.2	Reference	–	–	
Sexual behavior^a^								
Yes	195	0.2	271	0.1	2.0	1.7	2.5	<.0001
No	85,767	99.8	242,492	99.9	Reference	–	–	
Alcohol, drugs^a^								
Yes	975	1.1	781	0.3	3.6	3.3	4.0	<.0001
No	84,987	98.9	241,982	99.7	Reference	–	–	
Lifestyle^a^								
Yes	4,531	5.3	2,529	1.0	5.4	5.1	5.7	<.0001
No	81,431	94.7	240,234	99.0	Reference	–	–	
Life management^a^								
Yes	4,537	5.3	2,558	1.1	5.3	5.0	5.6	<.0001
No	81,425	94.7	240,205	98.9	Reference	–	–	
Victim of physical, sexual, psychological abuse^a^								
Yes	9,998	11.6	2,521	1.0	13.7	13.1	14.4	<.0001
No	75,964	88.4	240,242	99.0	Reference	–	–	
Victim of assault^a^								
Yes	654	0.8	507	0.2	3.7	3.3	4.2	<.0001
No	85,308	99.2	242,256	99.8	Reference	–	–	
Perpetrator of violence^a^								
Yes	108	0.1	50	0.0	5.8	4.2	8.2	<.0001
No	85,854	99.9	242,713	100.0	Reference	–	–	

Abbreviations: No., number; OR, odds ratio; LL, lower limit; UL, upper limit.

^a^Diagnosis within preceding 365 days.

**Table 4:** Adjusted Odds of Incident Suicidal Ideation or Attempt, Active Component U.S. Service Members with No History of Mental Health Diagnoses, 2018–2022

	Cases	Controls				
	No.	%	No.	%	OR	95% LL	95% UL	*p*-value
Education and literacy^b^								
Yes	0	0.0	0	0.0	N/A^a^			
No	3,204	100.0	9,239	100.0	Reference	–	–	
Employment^b^								
Yes	490	15.3	34	0.4	56.0	37.5	83.8	<.0001
No	2,714	84.7	9,205	99.6	Reference	–	–	
Housing, economic^b^								
Yes	4	0.1	0	0.0	N/A^a^			
No	3,200	99.9	9,239	100.0	Reference	–	–	
Social environment^b^								
Yes	375	11.7	33	0.4	35.9	24.7	52.1	<.0001
No	2,829	88.3	9,206	99.6	Reference	–	–	
Family upbringing^b^								
Yes	234	7.3	50	0.5	15.5	11.2	21.4	<.0001
No	2,970	92.7	9,189	99.5	Reference	–	–	
Other psychosocial^b^								
Yes	190	5.9	49	0.5	11.5	8.4	15.9	<.0001
No	3,014	94.1	9,190	99.5	Reference	–	–	
Sexual behavior^b^								
Yes	0	0.0	6	0.1	N/A^a^			
No	3,204	100.0	9,233	99.9	Reference	–	–	
Alcohol, drugs^b^								
Yes	3	0.1	9	0.1	1.0	0.3	3.7	1.0000
No	3,201	99.9	9,230	99.9	Reference	–	–	
Lifestyle^b^								
Yes	43	1.3	30	0.3	4.2	2.6	6.7	<.0001
No	3,161	98.7	9,209	99.7	Reference	–	–	
Life management^b^								
Yes	125	3.9	23	0.2	16.6	10.5	26.1	<.0001
No	3,079	96.1	9,216	99.8	Reference	–	–	
Victim of physical, sexual, psychological abuse^b^								
Yes	42	1.3	14	0.2	8.3	4.5	15.3	<.0001
No	3,162	98.7	9,225	99.8	Reference	–	–	
Victim of assault^b^								
Yes	3	0.1	13	0.1	0.6	0.2	2.3	0.4787
No	3,201	99.9	9,226	99.9	Reference	–	–	
Perpetrator of violence^b^								
Yes	1	0.0	1	0.0	3.0	0.2	48.0	0.4787
No	3,203	100.0	9,238	100.0	Reference	–	–	

Abbreviations: No., number; OR, odds ratio; LL, lower limit; UL, upper limit.

^a^Odds ratio could not be calculated due to small number of cases or controls.

^b^Diagnosis within preceding 365 days.

## AUTHOR AFFILIATIONS

Epidemiology and Analysis Branch, Armed Forces Health Surveillance Division, Public Health Directorate, Defense Health Agency, Silver Spring, MD: Dr. Ying, Dr. Mabila, Dr. Stahlman; Uniformed Services University of the Health Sciences, Bethesda, MD: Dr. Finlay
